# Supplementary material for: Cryo-EM structures of recombinant human sodium-potassium pump determined in three different states
Source: Nat Commun. 2022 Jul 8;13:3957. doi: 10.1038/s41467-022-31602-y (PMC9270386; doi:10.1038/s41467-022-31602-y)
Supplement: Supplementary file 1 — Supplementary Information [file 41467_2022_31602_MOESM1_ESM.pdf]

Supplementary Information

Cryo-EM structures of recombinant human sodium-potassium pump determined in three different states

Yingying Guo[1,2,3], Yuanyuan Zhang[1,2,3], Renhong Yan[1,2,3], Bangdong Huang[1,2], Fangfei Ye[1,2], Liushu Wu[1,2], Ximin Chi[1,2], Yi shi[1,2] and Qiang Zhou[1,2*]

[1]Westlake Laboratory of Life Sciences and Biomedicine, Key Laboratory of Structural Biology of Zhejiang Province, School of Life Sciences, Westlake University, 18 Shilongshan Road, Hangzhou 310024, Zhejiang Province, China.

[2]Institute of Biology, Westlake Institute for Advanced Study, 18 Shilongshan Road, Hangzhou 310024, Zhejiang Province, China.

[3]These authors contributed equally to this work.

*To whom correspondence should be addressed: zhouqiang@westlake.edu.cn (Q.Z.)

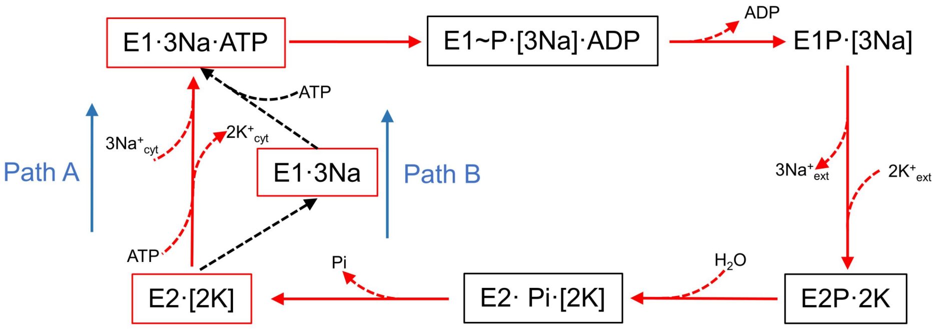

Supplementary Fig. 1 A simplified Post-Albers cycle for NKA. Previously determined structures are available for three reaction intermediates (black boxes). Red boxes mark structures for E1·3Na, E1·3Na·ATP and E2·[2K] are described in this study. The cycle marked in red lines represent the physiological mode, and black dashed lines represent the unphysiological mode. Pi, inorganic phosphate. E1P/E2P indicate the phosphoenzyme intermediates. Brackets represent occluded states, and cyt and ext are short form for cytosolic and extracellular, respectively.

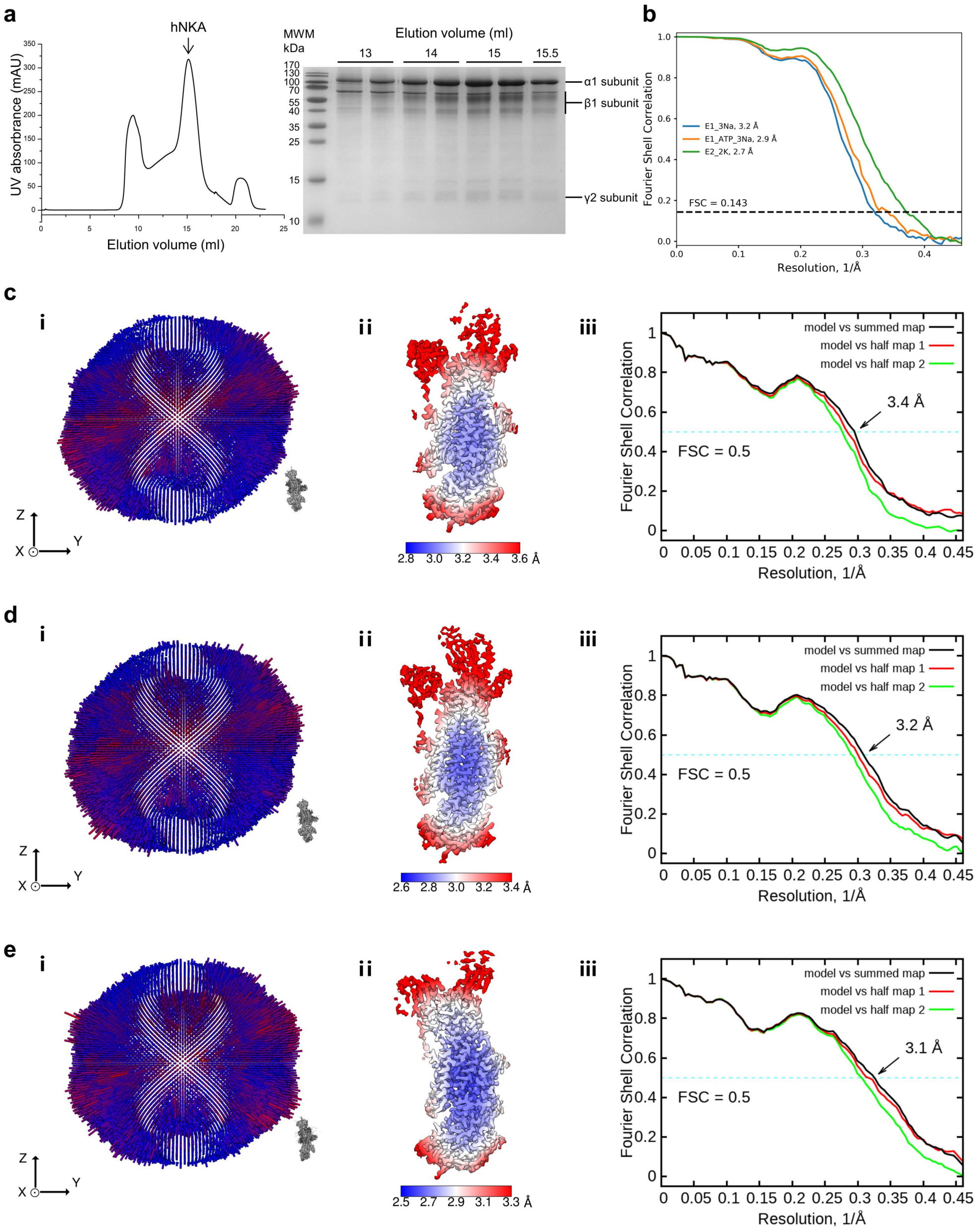

Supplementary Fig. 2 Cryo-EM analysis of three hNKA states. a, SEC purification of hNKA in the presence of 0.02% GDN and 0.001% cholesteryl hemisuccinate (CHS). Left, SEC chromatogram; Right, SEC fractions were subjected to SDS–PAGE and visualized by Coomassie blue staining. The peak fractions (14.5-15 ml) were collected and concentrated for EM analysis. Purification experiments were repeated at least three times for each hNKA states. **b,** Gold standard FSC curve of the overall structure of E1·3Na (blue), E1·3Na·ATP (orange) and E2·[2K] (green), respectively. **c** is for E1·3Na, **d** is for E1·3Na·ATP and **e** is for E2·[2K]. **i,** Euler angle distribution in the final 3D reconstruction. **ii ,** Local resolution map for the 3D reconstruction of

whole map. iii, FSC curve of the refined model versus the whole structure that it is refined against (black); of the model refined against the first half map versus the same map (red); and of the model refined against the first half map versus the second half map (green). The small difference between the red and green curves indicates that the refinement of the atomic coordinates did not suffer from overfitting.

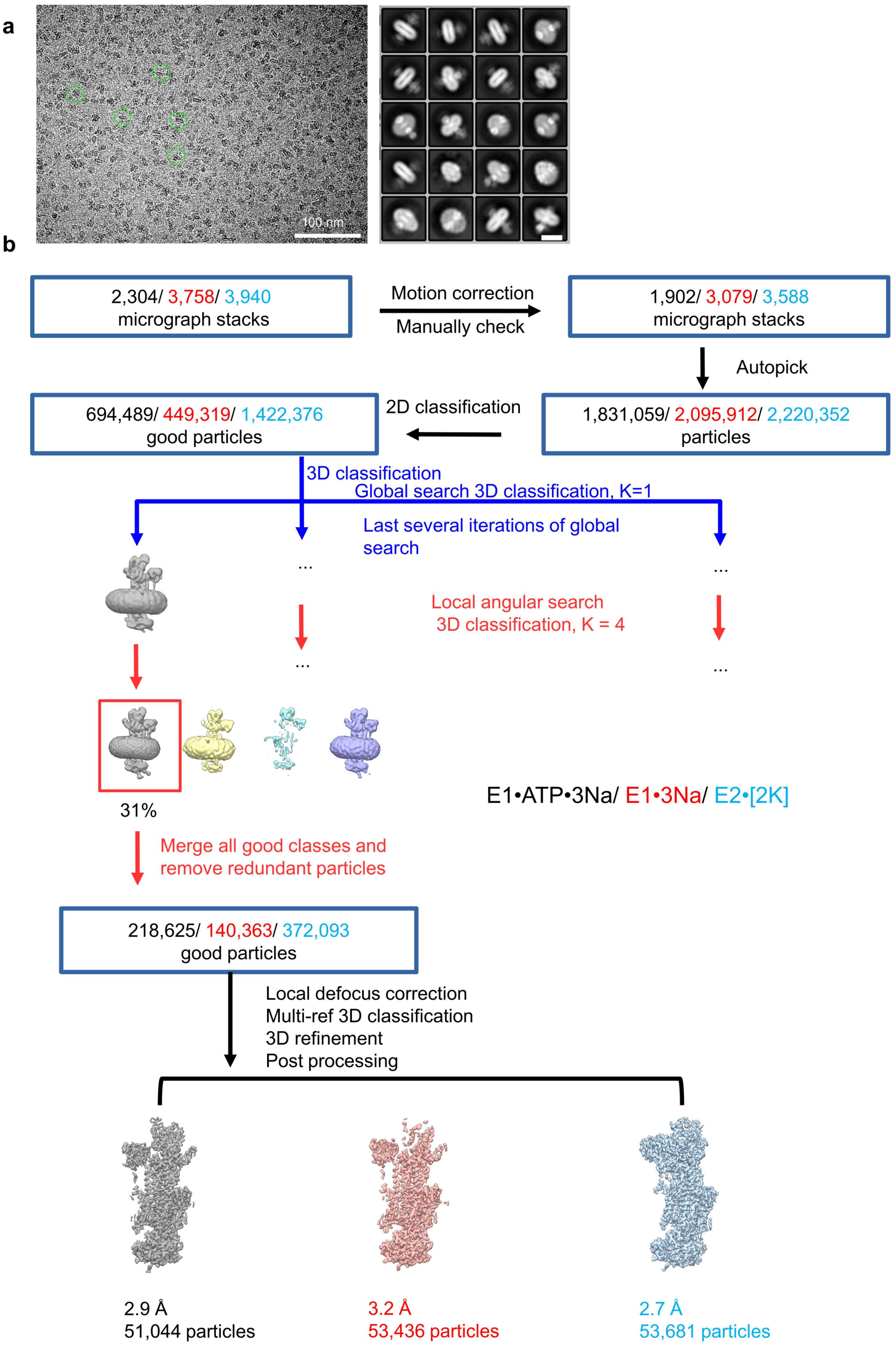

Supplementary Fig. 3 Cryo-EM data processing. a, Representative motion-corrected micrograph (Scale bar 100 nm) and 2D class averages of E1·3Na·ATP. The scale bar in 2D class averages (right panel) is 10 nm. The raw images are similar for all three samples. **b,** Flow chart of Cryo-EM data processing, please see the 'Data Processing' in Methods section for details.

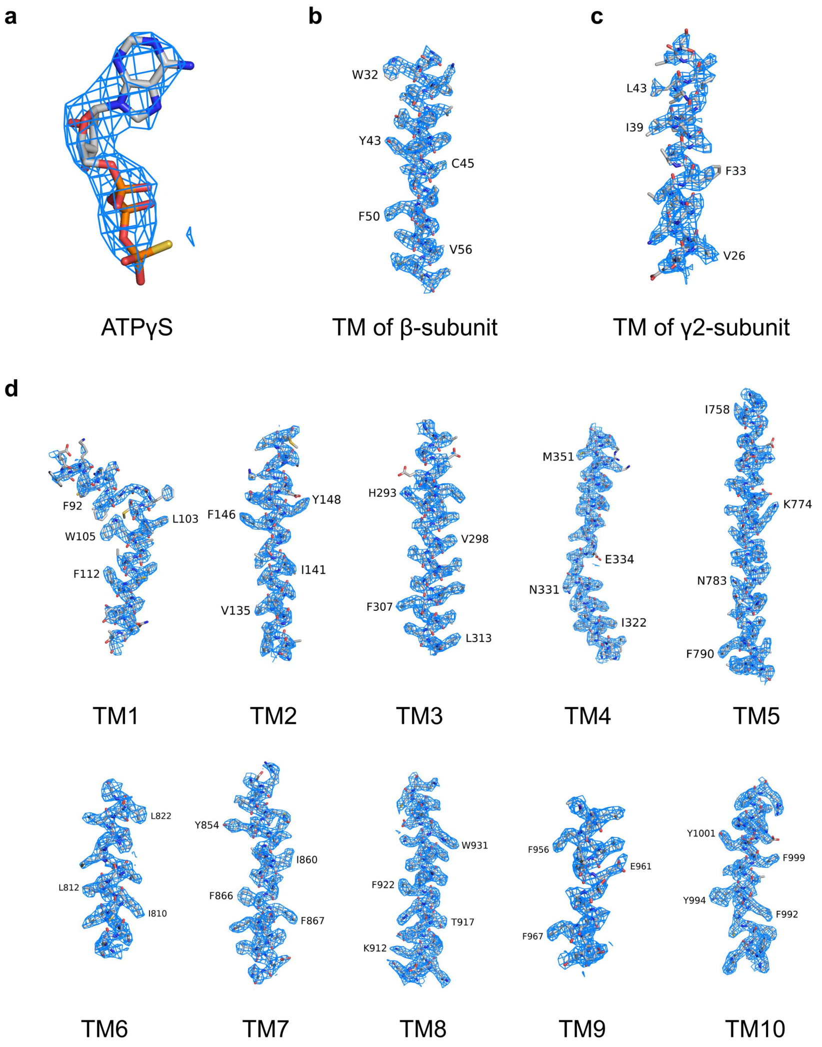

Supplementary Fig. 4 Cryo-EM density maps. a, Cryo-EM density map for ATPγS binding in the N domain pocket shown at threshold of 6 σ. **b,** Cryo-EM density map of the only TM helix of β1-subunit shown at threshold of 6 σ. **c,** Cryo-EM density map of the only TM helix of γ2-subunit shown at threshold of 4 σ. **d,** Cryo-EM density maps for the TM helices of α-subunit shown at threshold of 6 σ.

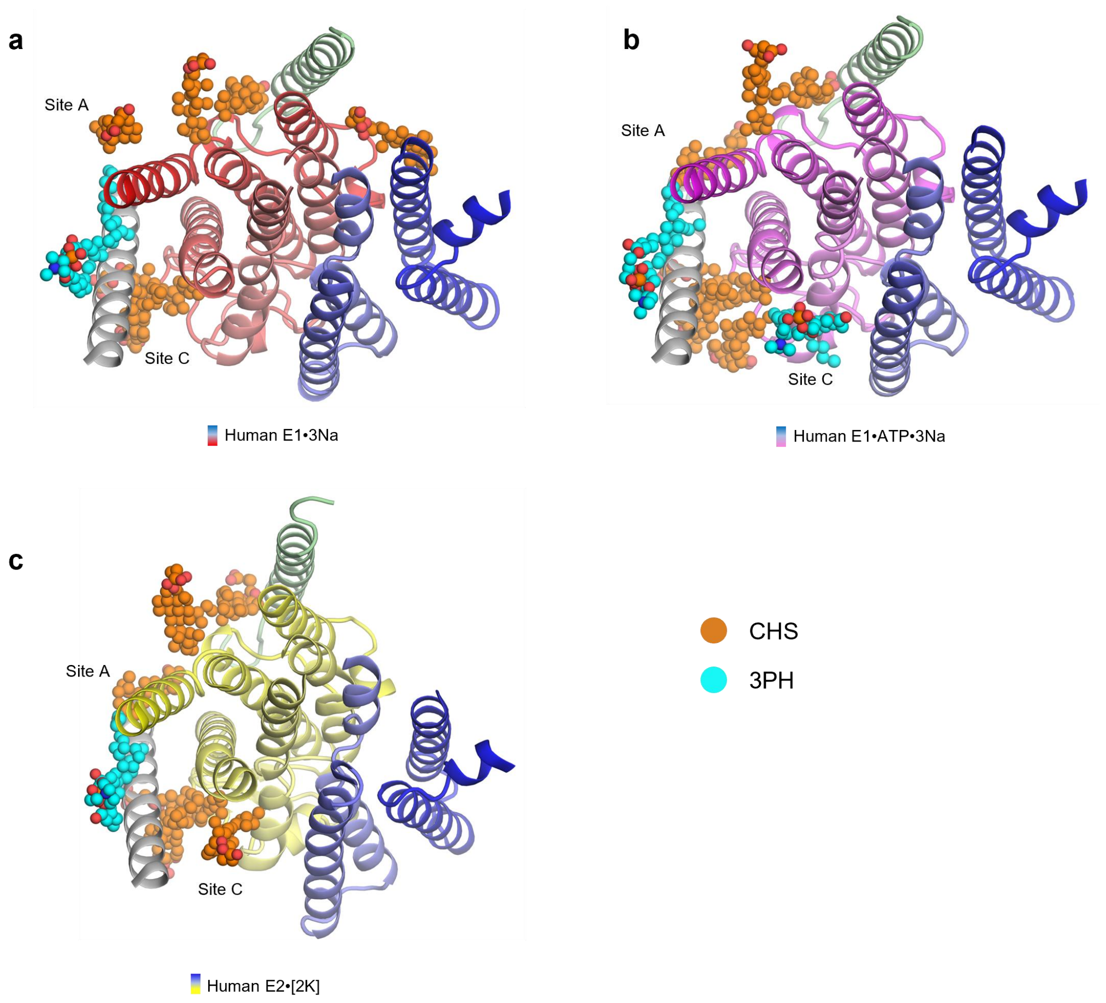

Supplementary Fig. 5 Lipids in E1·3Na, E1·3Na·ATP and E2·2[K] state. a-c, Cross-sectional area of hNKA near the membrane interface at cytoplasmic side of E1·3Na, E1·3Na·ATP and E2·2[K]. CHS and 3PH are colored orange and cyan, respectively.

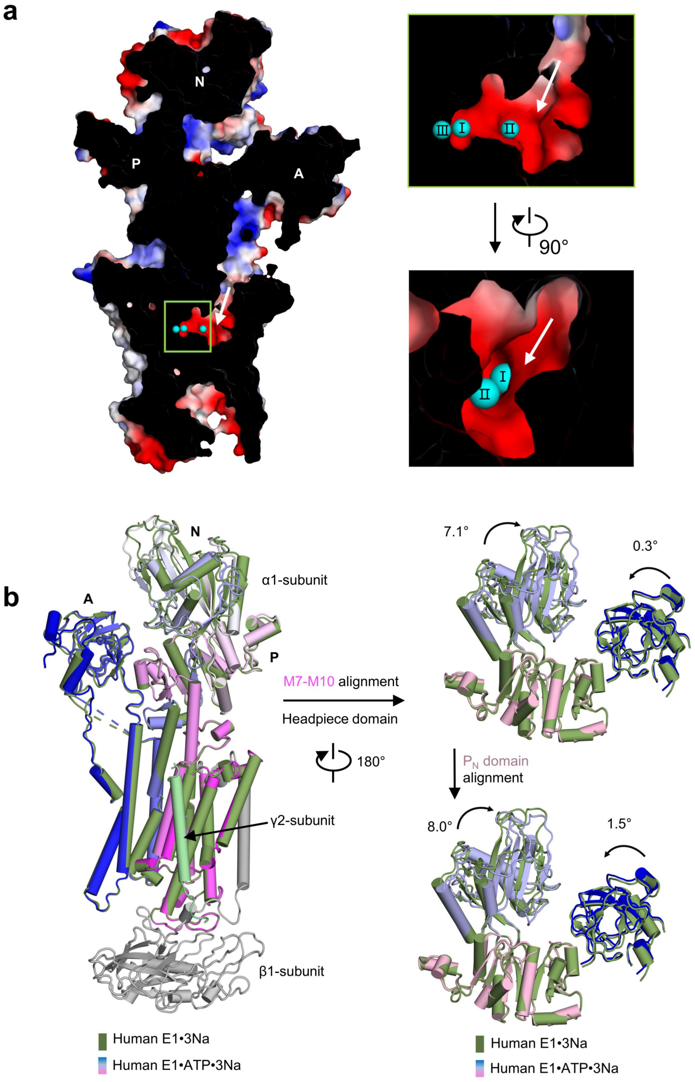

Supplementary Fig. 6 Sodium ion access path and structural comparison. a, Cross-section through the Na^+ entry pathway in E1·3Na state. Surface representation of hNKA α1 subunit is colored by vacuum electrostatics. Na^+ are colored cyan. **b,** Conformational changes between the E1·3Na (dark green) and the E1·3Na·ATP (blue to magenta).

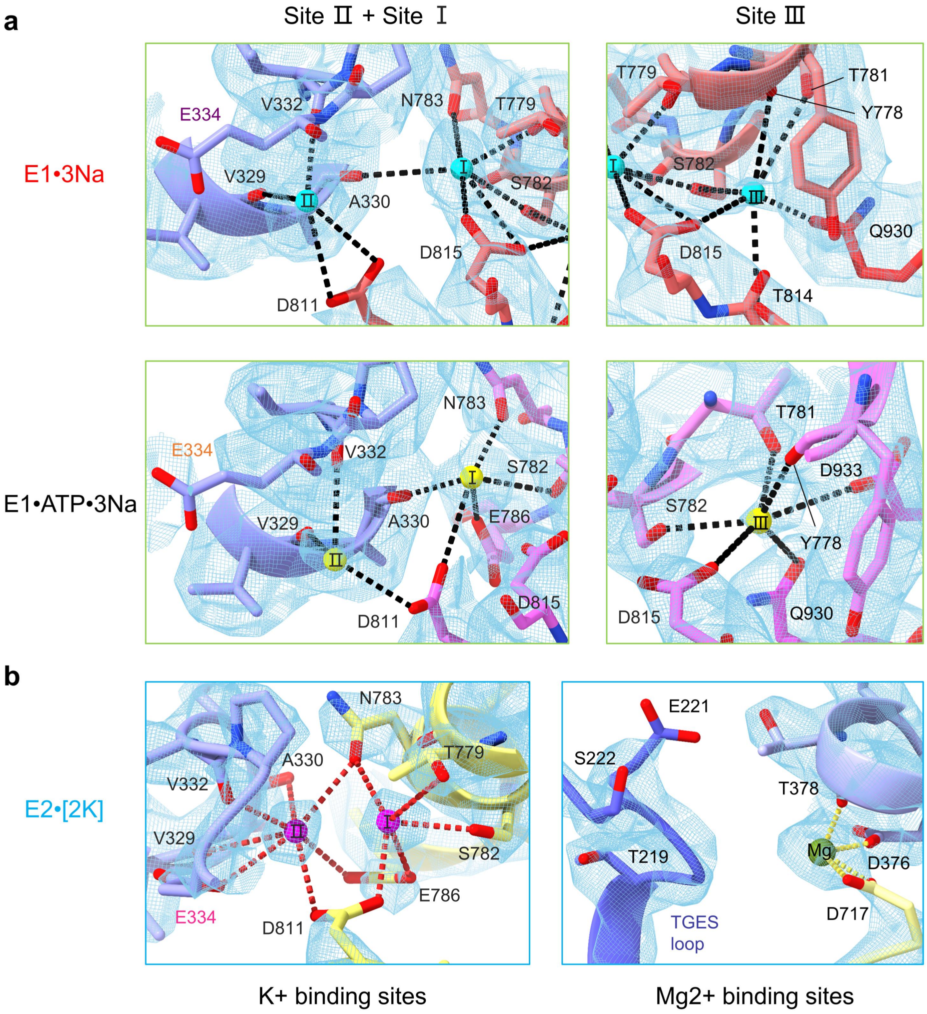

Supplementary Fig. 7 Cation binding sites. a, b Atomic models and corresponding cryo-EM density maps near cation binding sites in E1·3Na (blue mesh, 3 σ), E1·3Na·ATP (blue mesh, 3 σ), and E2·[2K] (blue mesh, left: 8 σ, right: 6 σ) state.

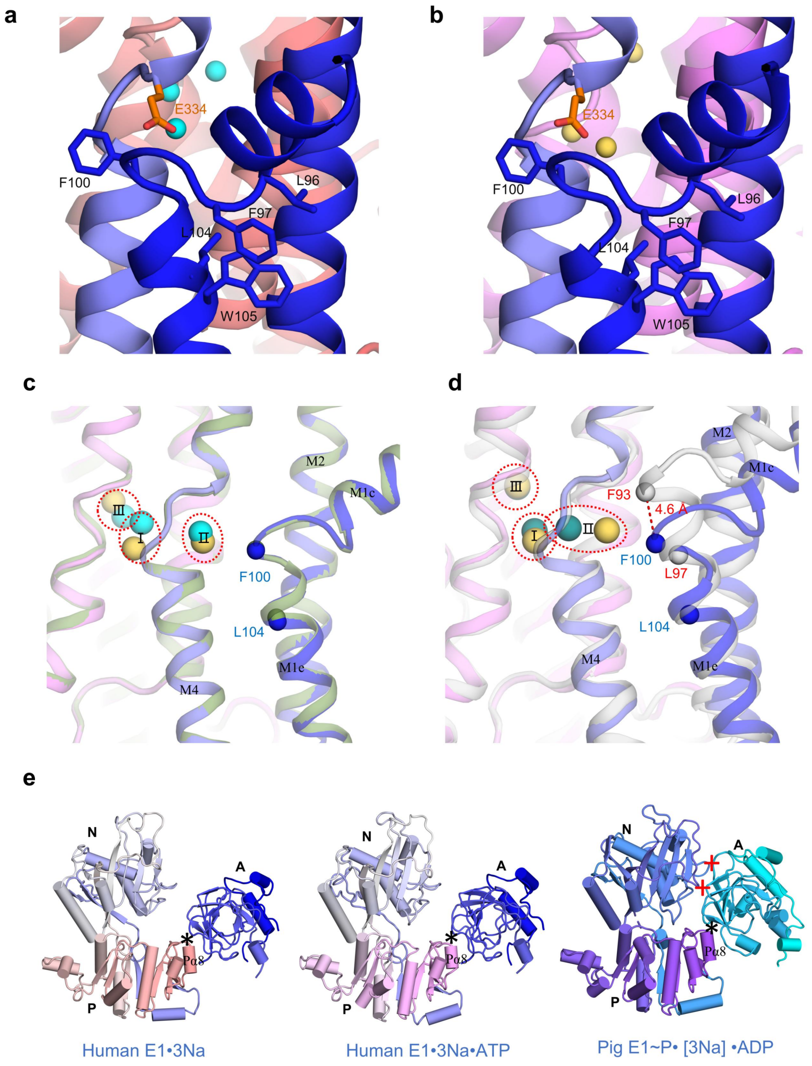

Supplementary Fig. 8 Structural comparison among the E1·3Na, E1·3Na·ATP and E1~P·[3Na]·ADP states. a, b Some amino acids around E334 in E1·3Na (blue to red) and E1·3Na·ATP (blue to magenta). **c,** The M1e is nearly identical between E1·3Na (dark green) and E1·3Na·ATP (blue to magenta). However, locations of Na^+ are different between them. **d,** Compared with E1~P·[3Na]·ADP (grey), the M1e of the E1·3Na·ATP shifts towards the extracellular side for about 4.6 Å. **e,** The open cytoplasmic headpiece are shown in E1·3Na (blue to red) and E1·3Na·ATP (blue to magenta), while the closed cytoplasmic headpiece is shown in E1~P·[3Na]·ADP (cyan to magenta).

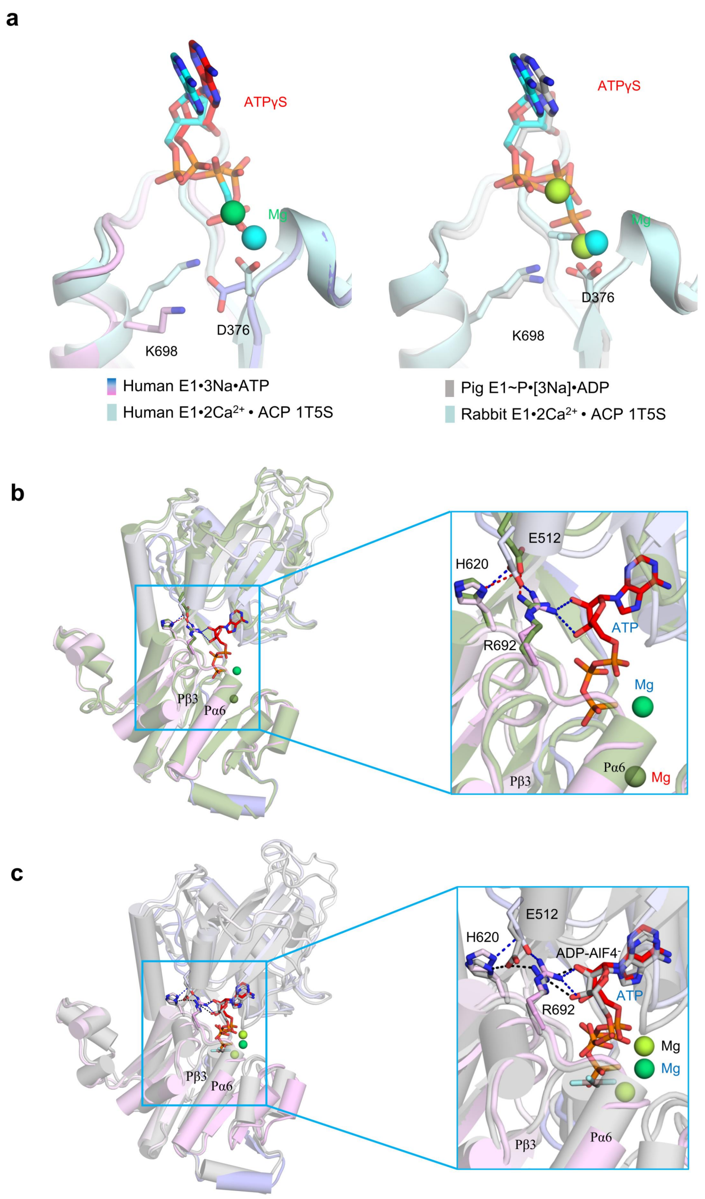

Supplementary Fig. 9 Conformational changes between the E1·3Na and E1·3Na·ATP states. a, Comparison of the Mg^{2+} binding site and the phosphorylation site (Asp376) between E1·$2Ca^{2+}$·ACP (pale cyan) and E1·3Na·ATP (blue to magenta) or E1~P·[3Na]·ADP (grey). **b, c** Comparing E1·3Na, E1·3Na·ATP and E1~P·[3Na]·ADP, Arg692 in P domain is a conserved pivot. Arg692 interacts with Glu512 of N domain in three states, also interacts with ATP in E1·3Na·ATP states, which pivot N domain tilt.

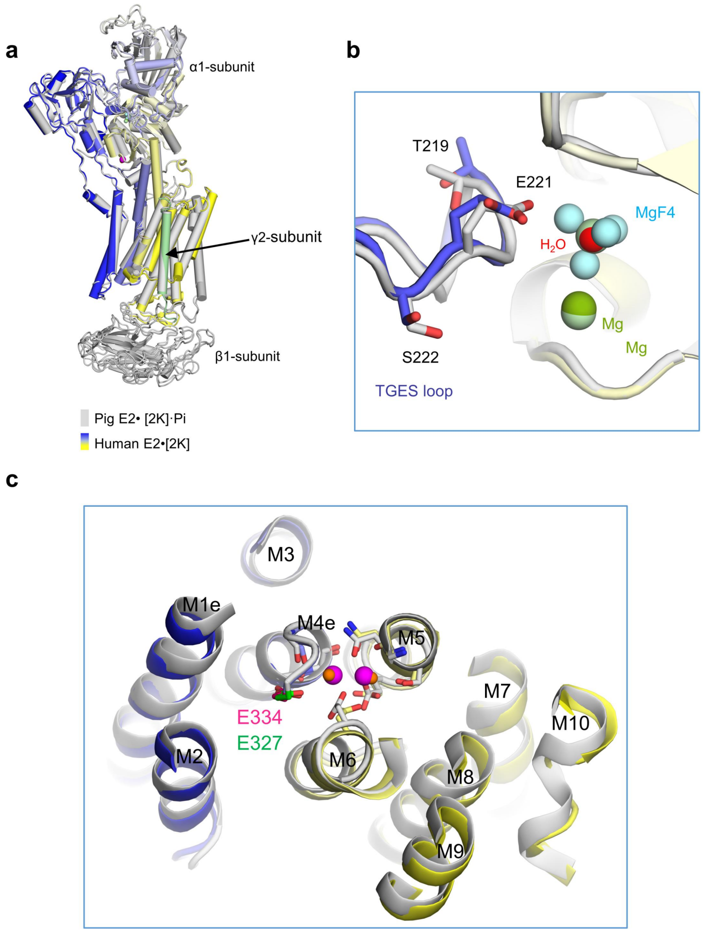

Supplementary Fig. 10 Structural comparison between the E2·[2K] and E2·[2K]·Pi states. **a,** Superposition between E2·[2K] (blue to yellow) and E2·[2K]·Pi (grey, PDB ID: 3KDP). **b,** The hallmark ^{219}TGES motif in the A domain is located near Mg^{2+} sites. **c,** Disposition of the transmembrane helices and K^+ binding sites of E2·[2K] and E2·[2K]·Pi viewed from the cytosolic side, approximately perpendicular to the membrane.

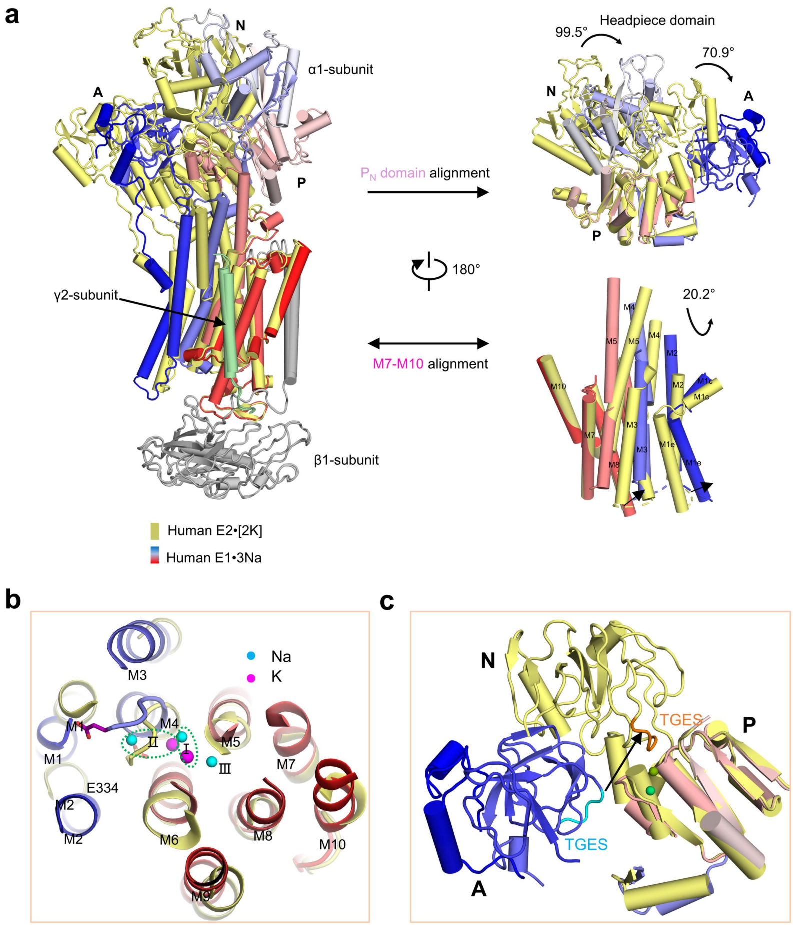

Supplementary Fig. 11 Structural comparison between E2·[2K] and E1·3Na states. a, Superposition between E2·[2K] (blue to yellow) and E1·3Na (blue to red). **b,** Disposition of the transmembrane helices and cations binding sites of E2·[2K] and E1·3Na viewed from the cytosolic side, approximately perpendicular to the membrane. **c,** The [219]TGES motif of A domain has largely movement to expose the conserved phosphorylation site in E1·3Na.

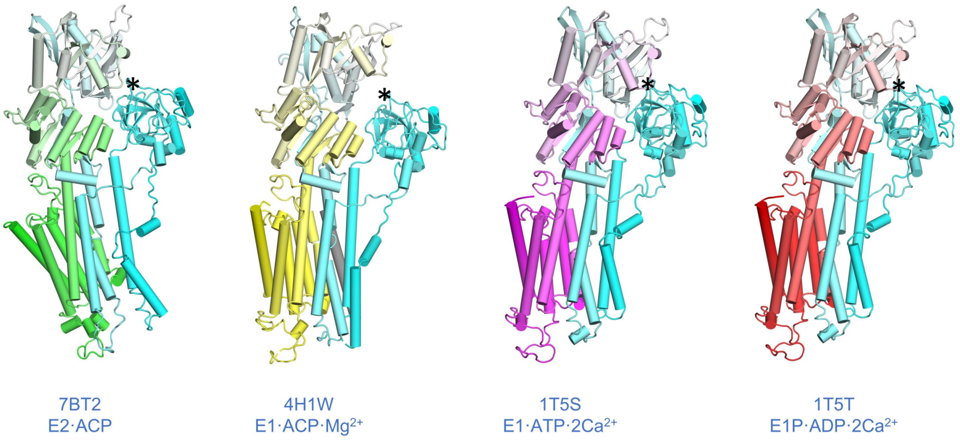

Supplementary Fig. 12 Structural comparison of SERCA among E2·ACP and E1 states. Asterisks indicate movement of A domain during Post-Albers cycle.

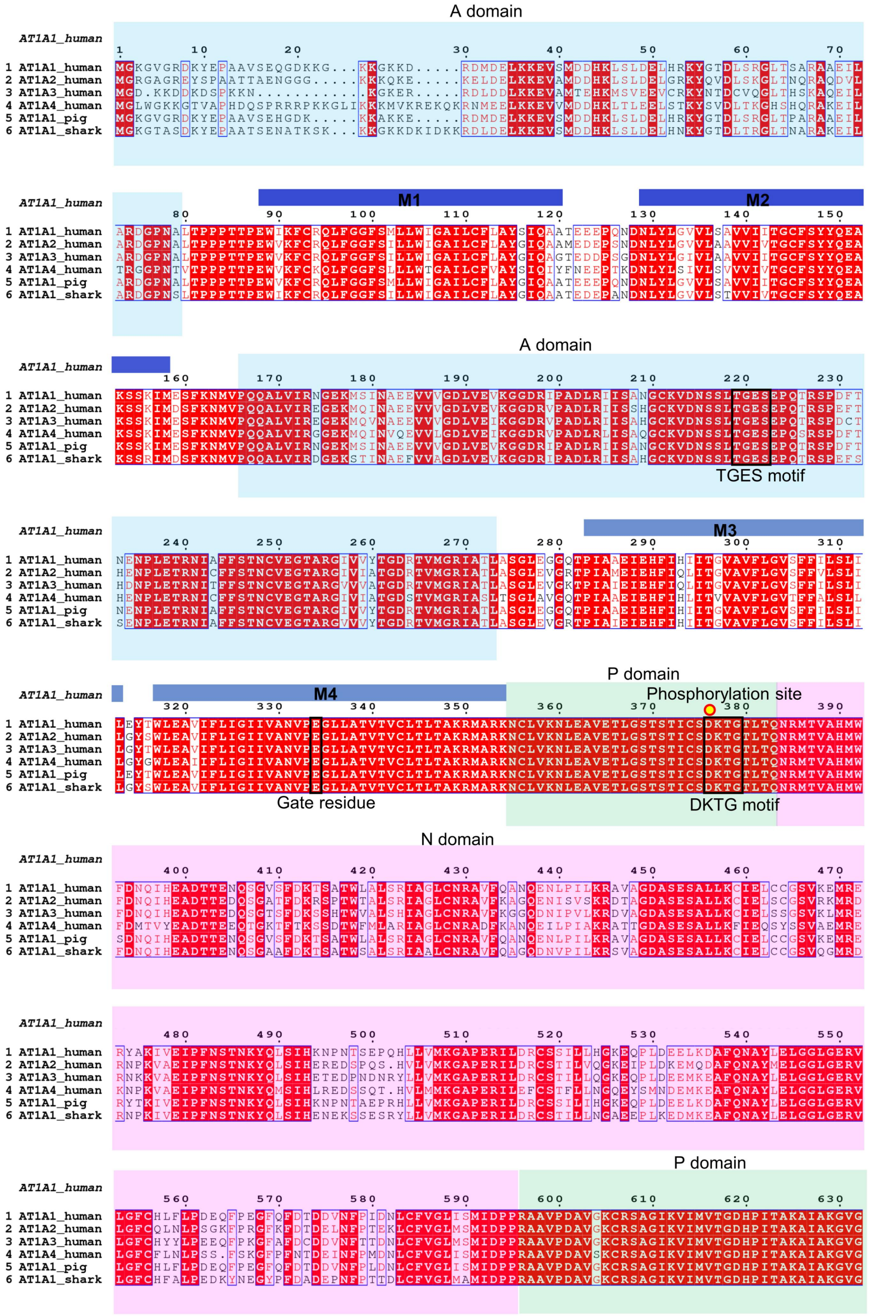
A domain
M1
M2
A domain
TGES motif
M3
P domain
Phosphorylation site
M4
Gate residue
DKTG motif
N domain
P domain
1 AT1A1_human
2 AT1A2_human
3 AT1A3_human
4 AT1A4_human
5 AT1A1_pig
6 AT1A1_shark

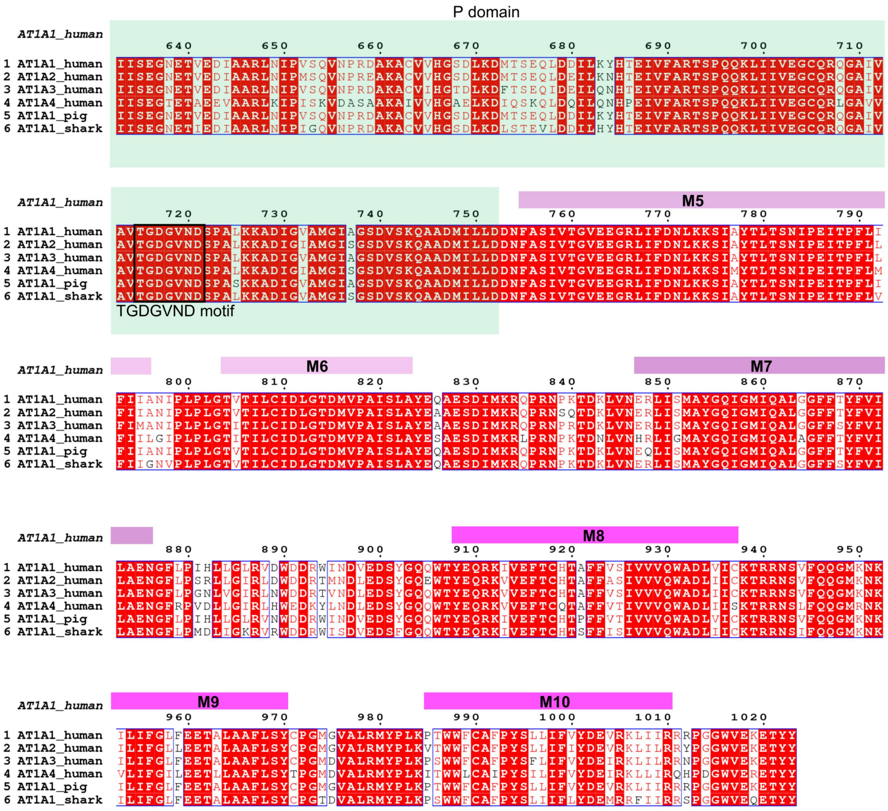

Supplementary Fig. 13 Sequence alignment of NKA. α subunit of four human isoforms, pig isoform1 and shark isoform1. The hallmark motifs and the gating residue are shown by black boxed. Phosphorylation site is indicated by yellow cycle. Various colors are used to distinguish the cytoplasmic domain (A, light blue; P, light green; N, light pink). Gene and protein ID are as follows: AT1A1_human (Genebank: BAA00061.1), AT1A2_human (Genebank: AAA51797.1), AT1A3_human (Genebank: AAA51798.1), AT1A4_human (Genebank: AAQ07964.1), AT1A1_pig (Genebank: CAA27576.1), AT1A1_shark (Genebank: AJ781093.1). Sequences were aligned by CLUSTAL 2.1.

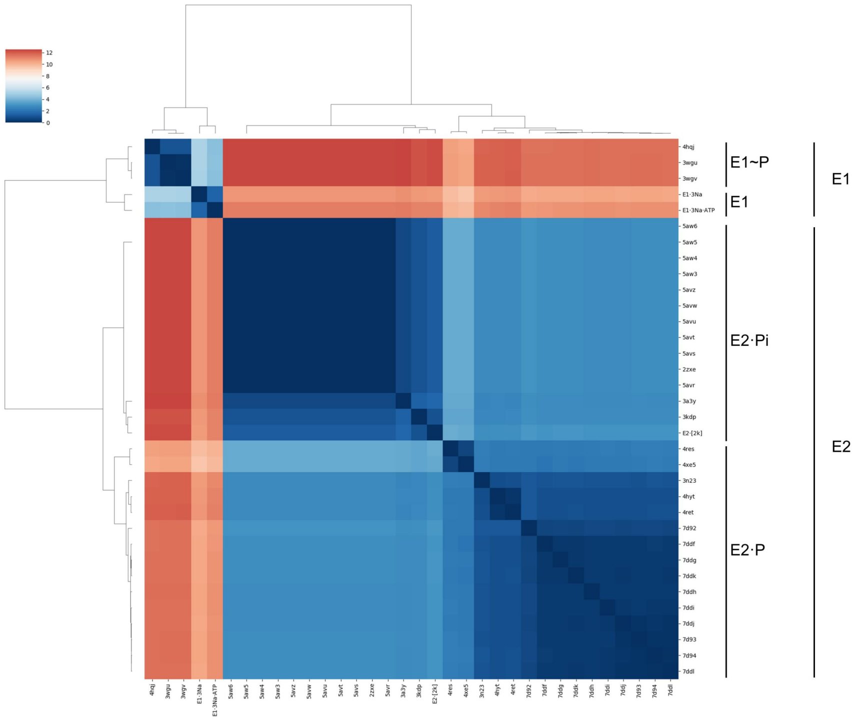

Supplementary Fig. 14 Cluster analysis of NKA structures. Paired RMSD was computed for all of 34 structures of NKA, according to which a heatmap was generated, revealing two major groups that represent the E1 and the E2 conformation, respectively. The E2 conformation has two subgroups: E2·P (including 15 structures) and E2·Pi (including 14 structures). The E1 conformation also contains two subgroups: E1>E1P (including 3 structures) and a subgroup consisting of the E1·3Na·ATP and the E1·3Na state reported in current work. The color codes correspond to RMSD (unit: Angstrom).

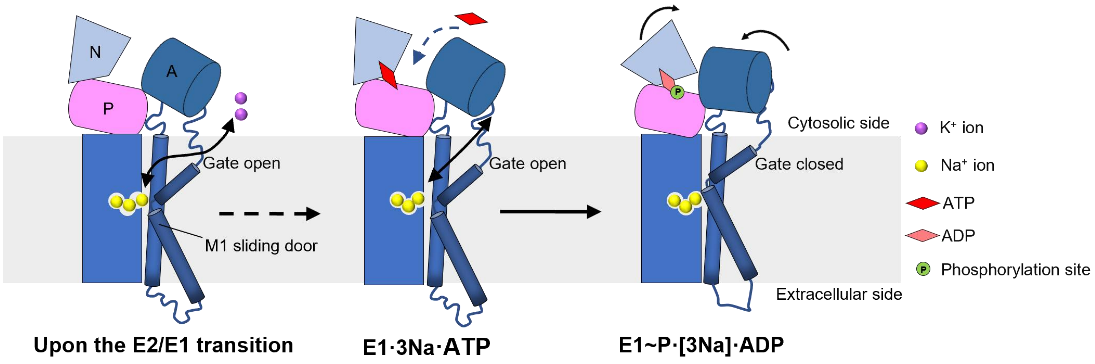

Supplementary Fig. 15 Schematic model of the cytosolic gating mechanism.

The first stage is gate open towards intracellular side after the transition from E2·[2K] to E1 states. Two K^+ were released into cytosol and three Na^+ were bound from intracellular side. ATP binding induces slightly tilt of the N domain and changes of the Na^+ binding sites, but the cytoplasmic gate remains open. After the ATP hydrolysis and phosphoryl transfer reaction, the rearrangement of three cytosolic domains forms a closed cytoplasmic headpiece. The movement and rotation of the A domain pull up the M1 helix after phosphorylation to close the cytoplasmic gate. The three cytosolic domains A, P and N are coloured marine, pink and light blue, respectively. The M1 sliding door linked with A domain is also coloured marine.

Supplementary Tables

Supplementary Table 1. Summary of data collection and model statistics for hNKA

Dataset	E1·ATP·3Na	E1·3Na	E2·[2K]
**Ligands**			
Na^{+}	150mM	150mM	−
K^{+}	−	−	100mM
Mg^{2+}	3mM	3mM	3mM
ATPγS	1mM	−	−
**Data collection**			
EM equipment		Titan Krios (Thermo Fisher Scientific)	
Voltage (kV)		300	
Detector		Gatan K3 Summit	
Energy filter		Gatan GIF Quantum, 20 eV slit	
Pixel size (Å)		1.087	
Electron dose (e-/Å^2)		50	
Defocus range (μm)		-1.2 ~ -2.2	
PDB code	7E21	7E1Z	7E20
EMDB code	EMD-30949	EMD-30947	EMD-30948
Number of collected micrographs	2,304	3,758	3,940
Number of selected micrographs	1,902	3,079	3,588
**3D Reconstruction**			
Software		Relion 3.0.6	
Number of used particles	51,044	53,436	53,681
Resolution (Å)	2.9	3.2	2.7
Symmetry		C1	
Map sharpening B-factor (Å^2)		-60	
**Refinement**			
Software		Phenix1.14	
Cell dimensions			
a=b=c (Å)		217.4	
α=β=γ (°)		90	
Model composition			
Protein residues	1,302	1,302	1,321
Side chains assigned	1,302	1,302	1,321
Sugar	3	3	3
ATPγS	1	0	0
Phospholipid	2	1	1
Cholesterol hemisuccinate	6	6	6
Na	4	4	0
K	0	0	3
Mg	1	1	1
Water	5	4	0

R.m.s deviations			
Bonds length (Å)	0.009	0.007	0.007
Bonds Angle (˚)	0.981	0.856	0.921
Ramachandran plot statistics (%)			
Preferred	91.42	92.58	93.16
Allowed	8.11	7.34	6.69
Outlier	0.46	0.08	0.15

Supplementary Table 2. The residues involved in Na^+ coordination within 4.0 Å distance and their partial valence contribution in E1·3Na state

Site	Residue	Atom	Distance(Å)	Valence	Total
I	Ala330	O	3.93	0.022	0.493
	Thr779	O	2.93	0.079	
	Ser782	O	3.96	0.022	
		OG	3.09	0.063	
	Asn783	OD1	3.46	0.039	
	Asp815	OD2	2.26	0.241	
	Asp815	OD1	3.78	0.027	
II	Val329	O	2.76	0.102	0.397
	Val332	O	2.63	0.126	
	Asp811	OD1	3.92	0.023	
		OD2	3.94	0.022	
	HOH1402	O	2.64	0.124	
III	Thr778	O	4.04	0.02	0.653
	Thr781	OG1	3.86	0.024	
	Ser782	OG	2.22	0.26	
	Thr814	OG1	3.82	0.025	
	Asp815	OD1	2.94	0.078	
	Gln930	OE1	2.25	0.246	

Supplementary Table 3 The residues involved in Na^+ coordination within 4.0 Å distance and their partial valence contribution in E1·3Na·ATP state

Site	Residue	Atom	Distance(Å)	Valence	Total
I	Ala330	O	2.34	0.208	0.748
	Ser782	O	3.54	0.035	
	Asn783	OD1	2.36	0.2	
	Glu786	OE1	2.63	0.126	
	Asp811	OD2	3.6	0.033	
	HOH1201	O	2.54	0.146	
II	Val329	O	2.58	0.137	0.313
	Val332	O	3.3	0.047	
	Asp811	OD1	3.28	0.049	
	HOH1202	O	3.77	0.027	
	HOH1204	O	3.83	0.025	
	HOH1205	O	3.72	0.028	
III	Thr778	O	2.54	0.416	0.717
	Thr781	OG1	2.76	0.102	
	Ser782	OG	3.03	0.069	
	Asp815	OD1	4.03	0.02	
	Gln930	OE1	2.93	0.079	
	Asp933	OD2	3.65	0.031	
