# Supplementary material for: Cryo-EM structures of recombinant human sodium-potassium pump determined in three different states
Source: Nat Commun. 2022 Jul 8;13:3957. doi: 10.1038/s41467-022-31602-y (PMC9270386; doi:10.1038/s41467-022-31602-y)
Supplement: Supplementary file 3 — Description of Additional Supplementary Files [file 41467_2022_31602_MOESM3_ESM.pdf]

File name: Supplementary Movie 1

Description: The structural morph between the open and closed gate conformation of NKA.
